# Non-canonical amino acid labeling in proteomics and biotechnology

**DOI:** 10.1186/s13036-019-0166-3

**Published:** 2019-05-22

**Authors:** Aya M. Saleh, Kristen M. Wilding, Sarah Calve, Bradley C. Bundy, Tamara L. Kinzer-Ursem

**Affiliations:** 1 Weldon School of Biomedical Engineering, Purdue University, West Lafayette, IN USA; 2 Department of Chemical Engineering, Brigham Young University, Provo, UT USA

**Keywords:** Metabolic labeling, Bioorthogonal chemistry, Residue-specific labeling, Site-specific labeling, Proteomics, Biotechnology

## Abstract

Metabolic labeling of proteins with non-canonical amino acids (ncAAs) provides unique bioorthogonal chemical groups during de novo synthesis by taking advantage of both endogenous and heterologous protein synthesis machineries. Labeled proteins can then be selectively conjugated to fluorophores, affinity reagents, peptides, polymers, nanoparticles or surfaces for a wide variety of downstream applications in proteomics and biotechnology. In this review, we focus on techniques in which proteins are residue- and site-specifically labeled with ncAAs containing bioorthogonal handles. These ncAA-labeled proteins are: readily enriched from cells and tissues for identification via mass spectrometry-based proteomic analysis; selectively purified for downstream biotechnology applications; or labeled with fluorophores for in situ analysis. To facilitate the wider use of these techniques, we provide decision trees to help guide the design of future experiments. It is expected that the use of ncAA labeling will continue to expand into new application areas where spatial and temporal analysis of proteome dynamics and engineering new chemistries and new function into proteins are desired.

## Overview of protein labeling with click chemistry functionality

Methods that allow for labeling of proteins co-translationally, i.e. as they are being synthesized, have wide ranging applications in engineering, biotechnology, and medicine. Incorporation of non-canonical amino acids (ncAAs) into proteins enables unique bioorthogonal chemistries, those that do not react with naturally occurring chemical functional groups, for conjugation. These conjugate substrates range from fluorophores, affinity reagents, and polymers to nanoparticle surfaces, enabling new advances in technology to study cellular systems and produce novel biocatalytic and therapeutic proteins. A key benefit of these techniques is the ability to enrich for labeled proteins of interest, whereas other labeling methods add or remove a mass (e.g. isotope labeling [1]) that can be difficult to identify when diluted within complex macromolecular mixtures. In this review, we focus specifically on techniques that incorporate click chemistry functionality into proteins of interest and provide decision tree analyses to guide selection of optimal strategies for protein labeling methods.

### Click chemistry functionality

First coined by Sharpless and colleagues in 2001, click chemistries are a set of chemical reactions that are readily catalyzed in aqueous solutions at atmospheric pressure and biologically-compatible temperatures, with few toxic intermediates, and relatively fast reaction kinetics [2]. The suite of specific click chemistry reactions that started with Staudinger ligation of azide and phosphine [3–5] and copper-catalyzed azide-alkyne cycloaddition [6, 7], has rapidly expanded to include more rapid and biologically friendly chemistries including strain promoted azide-alkyne cycloaddition [8, 9], oxime or hydrazine ligation [10, 11], strain-promoted alkyne nitrone cycloaddition [12, 13], tetrazine ligation [14, 15], and quadricyclane ligation [16, 17].

**Fig. 1:**
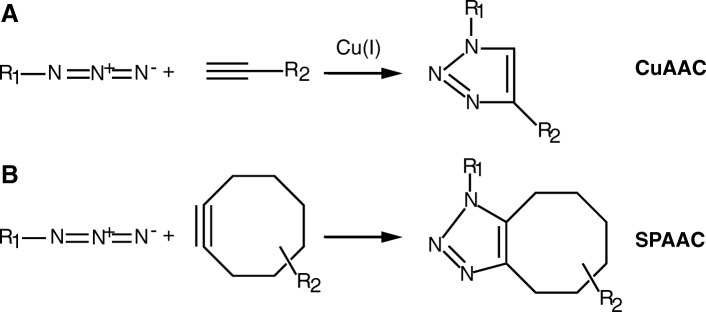
Azide-alkyne cycloaddition reactions. **a** Copper(I)-catalyzed [3 + 2] azide-alkyne cycloaddition (CuAAC). **b** [3 + 2] cycloaddition of azides and strain-promoted alkynes (cyclooctynes) (SPAAC)

Here, we focus on azide-alkyne cycloaddition as it is one of the most widely used, with broad availability of commercial reagents, moderately fast kinetics, and well-established protocols. Copper(I)-catalyzed azide-alkyne cycloaddition (CuAAC, Fig. 1a) has been implemented across disciplines, from biomaterials [18] and combinatorial chemistry [19] to polymer synthesis [20], protein activity tagging [21], and proteomics [22], some of which will be highlighted in later sections. One disadvantage of CuAAC is that there is significant cytotoxicity with using copper as the catalyst, hampering utilization in vivo [23]. To circumvent this limitation, Bertozzi and coworkers introduced a catalyst-free [3 + 2] cycloaddition reaction between azides and cyclooctyne derivatives, known as strain promoted azide-alkyne cycloaddition (SPAAC, Fig. 1b) [8, 23, 24]. The biocompatibility of this reaction was first demonstrated in Jurkat cells to label azide-tagged glycoproteins [8]. The strain-promoted azide-alkyne click reaction has since been applied in various in vivo settings with no apparent toxicity [24–27]. Importantly, CuAAC and SPAAC are bioorthogonal and will not interfere with natural biological chemistries.

### Labeling of nascent proteins

**Fig. 2:**
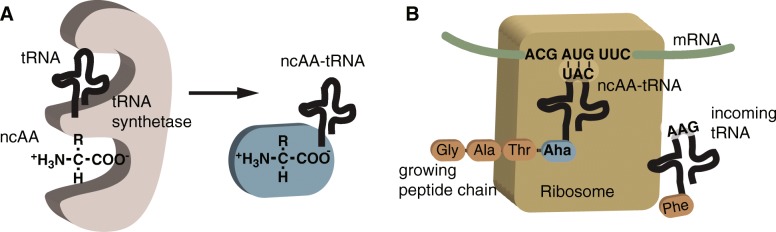
Incorporation of ncAAs by native cellular machinery. Non-canonical amino acids (ncAAs) are incorporated into the growing polypeptide chain as the protein is synthesized at the ribosome. **a** ncAA is covalently attached to a tRNA by aminoacyl tRNA synthetase (aaRS). **b** The tRNA, charged with the ncAA (ncAA-tRNA, ncAA in blue), recognizes mRNA codons in the ribosome and the ncAA is added to the growing polypeptide chain

Chemical biologists and bioengineers have found much utility in incorporating click chemistry functionality into nature’s translational machinery. In these methods, known as genetic code expansion or ncAA labeling [28–31], a ncAA carrying a desired click chemistry functional group is introduced to the host expression system and is incorporated onto an aminoacyl tRNA synthetase (aaRS) that covalently attaches the ncAA to the corresponding tRNA (Fig. 2a). The ncAA-tRNA complex is brought into the ribosome where the tRNA recognizes the appropriate mRNA codon sequence and the ncAA is added to the growing polypeptide chain (Fig. 2b). ncAA labeling can be designed to occur either at specific amino acid residues of interest, for example using a Methionine (Met) analog that carries an azide or alkyne functionality to replace any Met in a newly synthesized protein [3], or at specific sites in a protein of interest [32].

Though not the focus of this review, it is important to highlight other site-specific approaches for labeling proteins. These include leveraging of enzymatic post-translational modification of proteins with click-chemistry functionalized non-canonical fatty acids, nucleic acids, and sugars. These methods utilize so called ‘chemoenzymatic methods’ to label proteins at specific residues via enzymatic recognition of specific peptide sequences. In this way, endogenous, engineered, and recombinantly expressed proteins can be efficiently labeled in situ. Some examples include glycosylation [33–35], sortagging [36, 37] and fatty acylation [38–41], including prenylation [10, 42], palmitoylation [43, 44], and myristoylation [45–49].

#### Residue-specific labeling of nascent proteins with non-canonical amino acids

**Fig. 3:**
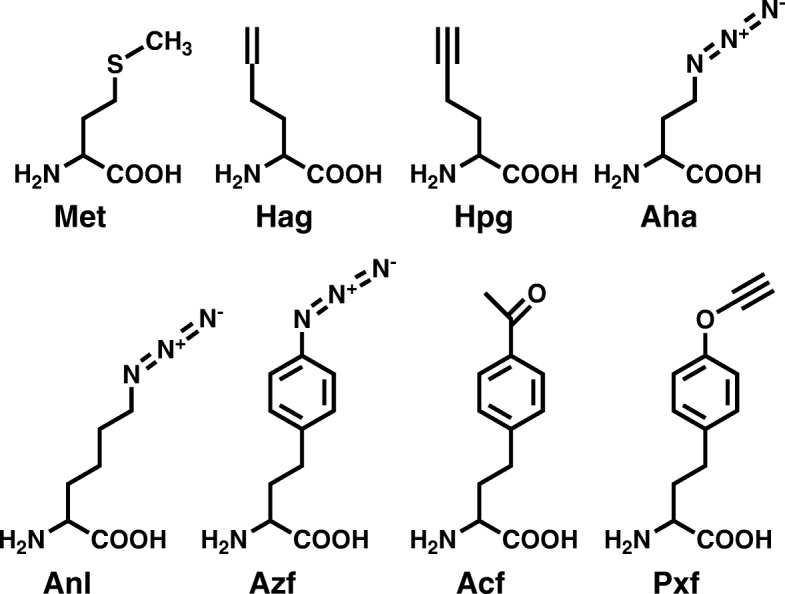
Examples of non-canonical amino acids. Chemical structures of amino acids highlighted in this review: methionine (Met), homoallylglycine (Hag), homopropargylglycine (Hpg), azidohomoalanine (Aha) and azidonorleucine (Anl). Azidophenylalanine (Azf), and acetylphenylalanine (Acf) are analogs of phenylalanine. Propargyloxyphenylalanine (Pxf) is a tyrosine analog (See Table 1 for more discussion of these ncAAs)

First demonstrated by Tirrell and colleagues, native translational machinery in *E. coli* was found to readily incorporate noncanonical Met analogs into proteins in vivo [50–52]. In this way, alkene (homoallylglycine, Hag) and alkyne (homopropargylglycine, Hpg) side-chain functionalities were added at Met sites during protein biosynthesis (Fig. 3, and Table 1). Later, azide analogs of Met (e.g. Aha, Fig. 3) were also found to be readily incorporated in vivo [3].

**Table 1:** List of ncAAs discussed in the review and their methods of incorporation

ncAA	Codon replacement	Natural amino acid analog	Genetic modification	Labeling method
Hag	AUG	Met	Not needed^a^	Residue-specific [50–52]
Hpg	AUG	Met	Not needed^a^	Residue-specific [22, 50, 51, 73]
Aha	AUG	Met	Not needed^a^	Residue-specific [3, 22, 75, 84–87]
Anl	AUG	Met	Transgenic lines that express a MetRS mutant capable of charging Anl^b^	Residue-specific [53, 54, 78, 88–90, 92–95]
Azf	UUC, UUU UAG	Phe	Transgenic lines that express a PheRS mutant capable of charging Azf^b^ Transgenic lines that express an orthogonal aaRS/amber suppressor tRNA pair evolved for Azf specificity	Residue-specific [55] Site-specific [118]
Acf	UUC, UUU UAG	Phe	Transgenic lines that express a PheRS mutant capable of charging Acf^b^ Transgenic lines that express an orthogonal aaRS/amber suppressor tRNA pair evolved for Acf specificity	Residue-specific [136] Site-specific [111]
Pxf	UAG	Tyr	Transgenic lines that express an orthogonal aaRS/amber suppressor tRNA pair evolved for Pxf specificity	Site-specific [124]

^a^The efficiency of the ncAA incorporation is greatly enhanced by using Met auxotrophic strains

^b^For cell selective labeling, the mutant aaRS is expressed under the control of cell-specific promoters

**Fig. 4:**
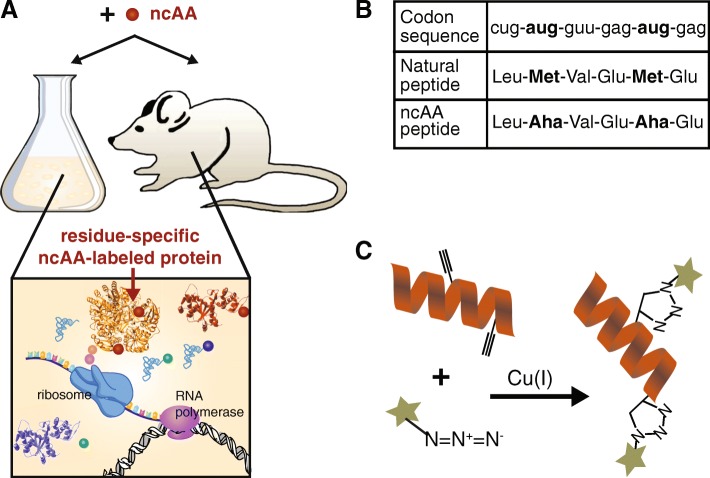
Overview of residue-specific protein labeling. **a** A ncAA (red sphere) is added to the system (cell culture or animal model). Native translational machinery incorporates the ncAA into the newly synthesized proteins. **b** An example of the codon sequence and corresponding peptides that result from either natural synthesis or synthesis in the presence of the ncAA. **c** A peptide labeled at two residue-specific sites with a ncAA carrying an alkyne functional group is conjugated to a azide-containing fluorophore via CuAAC

These methods take advantage of the ability for some ncAAs to incorporate (or become charged) onto native aaRSs (Fig. 2a), covalently attach to the corresponding tRNA, and subsequently incorporate into growing polpypeptide chains (Fig. 2b). The kinetics of Aha and Hpg binding to the methionyl tRNA synthetase (MetRS) are slower than that of Met (*k*_*cat*_*/K*_*m*_ of 1.42 × 10^− 3^ and 1.16 × 10^− 3^ s^− 1^·μM^− 1^ for Aha and Hpg, respectively vs 5.47 × 10^− 1^ s^− 1^·μM^− 1^ for Met) [3]. Nonetheless, this is a straightforward labeling method with no need for genetic engineering of the protein or organism under study (Fig. 4). For applications where 100% Met substitution is not necessary (e.g. enrichment for proteomics), adding the ncAA at concentrations where it can outcompete with Met provides sufficient functional incorporation. Alternatives that increase ncAA incorporation include using Met auxotrophic strains of *E. coli* that cannot make their own Met [52], or Met-free media for mammalian cell culture. Orthogonal aaRSs have also been engineered to bind to ncAAs in cells expressing the mutant aaRS, allowing for protein labeling with ncAAs in specific cell types [53–57].

#### Site-specific labeling of proteins with non-canonical amino acids

**Fig. 5:**
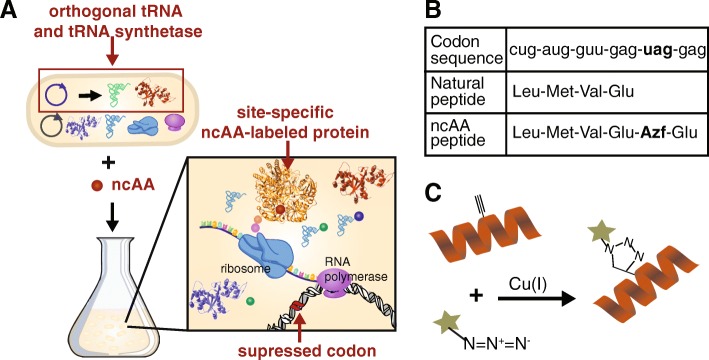
Overview of site-specific ncAA incorporation using orthogonal tRNA/aminoacyl synthetase pair. **a** A plasmid that expresses the desired orthogonal tRNA and tRNA synthetase is transfected into cells along with the plasmid containing the protein of interest that has been engineered to carry the suppressed codon sequence at a specific site. ncAA is added to the system and the protein of interest is labeled site-specfically with the ncAA. **b** An example of the codon sequence and corresponding peptides that result from either natural synthesis or synthesis in the presence of the orthogonal tRNA/tRNA synthetase and ncAA. **c** A peptide labeled site-specifically with a ncAA carrying an alkyne functional group is conjugated to a azide-containing fluorophore via CuAAC

An alternative to residue-specific ncAA incorporation is site-specific ncAA incorporation, in which a ncAA is incorporated exclusively at a pre-determined site. Motivated by the implications for detailed studying of protein structure and function, Schultz and colleagues were one of the first to demonstrate the feasibility of site-specific incorporation of ncAAs into a full-length protein in 1989 [32]. To accomplish this, the anticodon of suppressor tRNA molecules was engineered to recognize the amber stop codon (UAG), chemically aminoacylated with the ncAA, and then added to an in vitro protein synthesis system. Later, Furter site-specifically incorporated ncAAs in vivo by using an engineered orthogonal tRNA/tRNA synthetase pair for amber suppression. As illustrated in Fig. 5, the tRNA/tRNA synthetase pair is exogenous and operates orthogonally and the tRNA is specific for UAG instead of AUG [58]. Since then, over 100 different ncAAs have been incorporated either in vivo or in vitro in a variety of systems including bacteria, yeast, plant, mammalian, and human cells [59, 60]. The methods for site-specific ncAA incorporation have also expanded beyond amber codon suppression to include suppression of additional stop codons (nonsense suppression) [61, 62], recoding of sense codons [63], and recognition of 4-base codons (frameshift suppression) [62, 64, 65], though amber suppression is still the most widely used method.

As described above, initial ncAA incorporation was performed using chemically aminoacylated tRNA and an in vitro protein synthesis system [32, 65]. This method circumvents the need for evolving aaRSs to charge suppressor tRNA, and enables incorporation of virtually any ncAAs, including very large ncAAs such as those pre-conjugated to polyethylene glycol [64, 66]. Although chemically aminoacylated tRNA is still used for small-scale applications, it is not economically scalable for large-scale biotechnology applications, which must instead rely on enzymatic aminoacylation.

For large-scale applications, an orthogonal tRNA is engineered to recognize the specific codon sequence, and an orthogonal aaRS charges the engineered tRNA with the desired ncAA to enable continuous tRNA aminoacylation throughout protein expression (Fig. 5) [67]. The amber stop codon, UAG, is used less frequently by organisms than the other stop codons and is commonly targeted as the repurposed codon [68], though the other stop codons have also been successfully utilized [61, 62]. Frameshift suppression is executed similarly, by targeting a quadruplet codon [65]; however, suppression efficiencies are reportedly lower than nonsense suppression [62, 69]. By employing a combination of suppression techniques, multiple ncAAs can be site-specifically incorporated simultaneously [61, 62, 64, 69, 70]. In these cases, the suppression machinery must be mutually orthogonal in order to maintain site-specificity.

Overall, the site-specific approach provides significantly more control over the exact pre-defined location of ncAA incorporation into the protein as compared to other methods [71]. It also facilitates very high ncAA incorporation efficiencies [67]. As such, it is a powerful tool for biotechnology applications and will be detailed later in the paper. Potential uses of this technique for proteomics applications are still being developed and are briefly highlighted at the end of the following section.

## Applications of ncAA labeling

### Proteomics

#### Residue-specific labeling for proteomic applications

Residue-specific methods have since been applied to identify de novo protein synthesis in a variety of contexts. Dieterich et al. introduced the bioorthogonal non-canonical amino acid tagging (BONCAT) strategy for selective analysis of de novo protein synthesis with high temporal resolution [22, 72]. In this method, cells are cultured in media supplemented with Met analogs like Hpg or Aha, that are tagged with alkyne or azide functional groups, respectively (Fig. 4). Since azides and alkynes are bioorthogonal moieties, Hpg- and Aha-labeled proteins can be selectively conjugated to affinity tags even within complex cellular or tissue lysates to enrich the newly synthesized proteins from the pool of pre-existing unlabeled proteins. Additionally, labeled proteins can be ligated to fluorescent dyes for protein visualization using a sister technique referred to as fluorescent non-canonical amino acid tagging (FUNCAT) [25, 73].

Over the last decade, BONCAT has gained wide recognition because of its capability of tracking continuous changes in protein expression. It has been applied in mammalian cell cultures to study protein acylation [74], lysosomal protein degradation [75], and inflammation [76]. The method has also been used in various bacterial systems in order to explore quorum sensing [77], identify virulence factors [78], and monitor bacterial degradation in phagocytes [79]. Moreover, BONCAT has proven effective in more complicated biological systems such as zebrafish [80], *Caenorhabditis elegans* [55, 81], and Xenopus [82].

Until recently, it was presumed that BONCAT cannot be applied to in vivo labeling of the rodent proteome because mammalian cells would favor incorporating endogenous Met, rather than an analog, into newly expressed proteins [83]. However, Schiapparelli et al. successfully labeled newly synthesized proteins in the retina of adult rats by intraocular injection of Aha [84]. Further, McClatchy et al. showed that in vivo labeling of the entire murine proteome is feasible by feeding animals an Aha-enriched diet for 4 to 6 days [85, 86]. More recently, Calve and Kinzer-Ursem demonstrated that two days of intraperitoneal injection of Aha and Hpg is sufficient for systemic incorporation of the Met analogs into the proteome of both juvenile mice and developing embryos [87]. In this study, neither perturbation of physiological function of the injected mice nor atypical embryonic development was observed. In addition, both Aha and Hpg were successfully incorporated into different murine tissues in a concentration-dependent manner [87]. Notably, labeling with Hpg was less efficient than Aha, which is in agreement with findings by Kiick et al. that the activation rate of Hpg by MetRS is slower than Aha [3]. In light of these results, the successful incorporation of Met analogs into the entire murine proteome through intraperitoneal injection will pave the road for using animal models to temporally map protein expression. This method provides several advantages over introducing ncAAs via diet because intraperitoneal injection is relatively easy to perform, global proteome labeling is achieved in a shorter time period, and injection warrants more accurate dose-effect calculations.

With the aim of probing proteomic changes in specific cell types, engineered aaRS technology was adopted to allow cell-selective labeling with ncAAs. This technique, pioneered by the Tirrell group, was first made possible by identifying *E. coli* MetRS mutants that can charge the Met analog azidonorleucine (Anl) to Met sites [88]. Anl is not a substrate to endogenous aaRSs, hence only cells bearing the mutant MetRS are labeled. Since its discovery, the mutant MetRS Anl labeling technique has been applied to label nascent proteomes of *E. coli* [51, 57, 89, 90], *Salmonella typhimurium* [91], *Yersinia enterocolitica* and *Yersinia pestis* strains [78], and *Toxoplasma gondii* [92] in infected host cells. The exclusive expression of the mutant MetRS in these pathogens allowed selective detection of pathogen proteins among the more abundant host proteins.

To further demonstrate the utility of this approach, other variants of aaRS have been evolved to enable cell-selective incorporation of ncAAs in mammalian cells and animals. Using *Caenorhabditis elegans* as a model organism, Yuet et al. employed a phenylalanyltRNA synthetase mutant capable of incorporating the ncAA azidophenylalanine (Azf, Fig. 3) into worm proteins [55]. In their study, cell-type-specific resolution was achieved by expressing the mutant synthetase in targeted cells under the control of cell-specific promoters. Similarly, Erdmann et al. demonstrated that cell selectivity in *Drosophila melanogaster* can be achieved by using murine (mMetRS) and drosophila MetRS (dMetRS) mutants that can activate Anl [93]. The dMetRS variant was further utilized by Niehues et al. to study protein synthesis rates in a drosophila model of Charcot–Marie–Tooth neuropathy [94], while the mMetRS variant was applied to selectively tag astrocyte proteins in a mixed culture system [95] and to label the nascent proteome of several mammalian cells [54].

More recently, Schuman and coworkers advanced the MetRS mutant technology to enable cell-selective labeling in live mammals for the first time [53]. In this seminal work, selective labeling and identification of nascent proteomes in hippocampal excitatory and cerebellar inhibitory neurons was achieved using a transgenic mouse line wherein a MetRS mutant is expressed under the control of Cre recombinase. The Conboy group expanded this technique to identify the “young” proteins that were transferred to old mice in a model of heterochronic parabiosis [96]. This application was further leveraged by the Conboy and Aran groups by designing a graphene-based biosensor capable of selective capturing and quantifying of azide-labeled blood proteins that traveled from young to old parabiotic pairings [97], signifying the potential utility of cell-selective technology in the field of diagnosis and biomarker discovery.

#### Site-specific labeling for proteomic applications

**Fig. 6:**
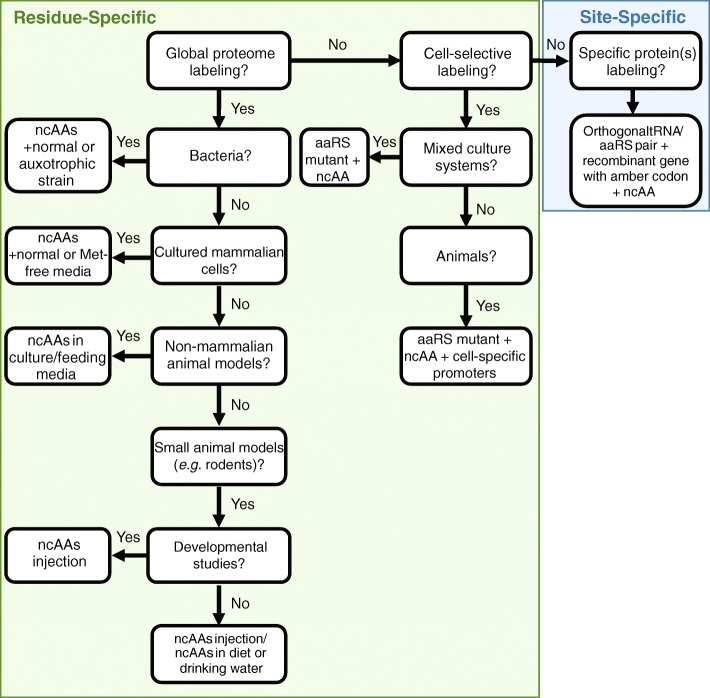
Decision tree for ncAA labeling in proteomics applications. If global proteome labeling is desired, consider residue-specific labeling. Residue-specific ncAA labeling is designed to replace a specific natural amino acid of interest in the entire proteome. Several natural amino acids analogs have been utilized (See Fig. 3 and Table 1). No genetic modification is needed for global proteome labeling with ncAA. Nevertheless, the labeling efficiency in bacterial cells is greatly enhanced if auxotrophic mutants are used. Similarly, labeling of cultured mammalian cells and non-mammalian animal models (e.g. nematodes) can be achieved by adding the ncAA directly to the culture/feeding media. However, if higher degree of labeling is required, consider using culture media that lacks the natural amino acid to be replaced. For in vivo labeling of small animal models (e.g. rodents), the ncAA can be injected or added to animal diet and/or drinking water. If embryonic labeling is desired, consider ncAA injection since it has been demonstrated that ncAAs are effectively incorporated into embryos when injected into pregnant animals without disturbing normal development [87]. If labeling of specific cell types in a mixed culture system is desired, consider using transgenic lines that express a mutant aaRS designed to charge the ncAA of interest. Since the ncAA is not a substrate of endogenous aaRSs, only cells expressing the mutant aaRS in the mixed culture system are labeled. Similarly, if cell-selective labeling of animals is required, consider use transgenic animals that express the mutant aaRS under cell-specific promoters. If specific protein labeling rather than global proteome labeling is needed, ncAA can be incorporated site-specifically in the polypeptide chain in response to an amber stop codon. This requires introducing the amber codon into the gene of interest and using an orthogonal aaRS/amber suppressor tRNA pair evolved for charging the desired ncAA

While residue-specific ncAA labeling has been primarily used for proteomic applications due to the ease of use and incorporation throughout the proteome, site-specific labeling has the potential to also assist in this area [53, 98]. For example, ncAAs could be used to label and trace a specific protein as it is expressed, migrates, and travels within a cell or tissue. In addition, ncAAs could be combined with proteomics to track a specific protein that is available at low levels. A factor that has limited site-specific ncAA use in proteomics is that this area of research has been focused on single-celled organisms, whereas proteomic studies are commonly performed in multicellular organisms. Recently, site-specific ncAA has been expanded to the multicellular organisms *Caenorhabditis elegans* and *Drosophila melanogaster* [99, 100], holding promise for the implementation into additional multicellular organisms*.* In the meantime, residue-specific labeling will continue to be the predominant approach when using ncAAs for most proteomics applications. With the increasing variety of approaches to ncAA incorporation, it is important to identify which approaches are best suited to a given application. To help guide researchers find the optimal strategy to label proteins for a given proteomics application, a decision tree diagram is provided in Fig. 6.

### Biotechnology applications

Traditional approaches to bioconjugation in biotechnology often target reactive side chains of natural amino acids such as lysines, though this results in a complex mixture of products modified at different locations and to different extents, complicating protein separation and often reducing protein activity. For some applications, sufficient control is afforded by altering the pH of a conjugation reaction to enhance the reactivity of the N-terminal amino group [101, 102]. An advantage of this method is circumvention of protein mutation, however, restricting bioconjugation to the N-terminus limits the potential for conjugation site optimization and can be deleterious to the structure and function, as was found to be the case with parathyroid hormone [101]. Surface-exposed cysteines, either native or substituted into the proteins, may also be targeted for modification, as they are more limited than other reactive amino acids such as lysine [103]. However, successful application of these methods is limited by the inherent properties of the target protein – in some proteins, the N-terminus may be inaccessible or involved in protein function, and engineering cysteine sites into proteins with natural cysteines may interfere with native disulfide bond formation.

As uniquely reactive chemical moieties, ncAAs provide a tool for enhancing commercial and therapeutic applications of proteins in a bioorthogonal manner. ncAAs have been used to study protein stability and also generate proteins with improved stability [104]. Characterization of protein structure and conformation, properties essential for effective rational enzyme and drug design, can also be improved through FRET analysis following conjugation of fluorophores to incorporated ncAAs [63, 105, 106]. Click chemistry compatible ncAAs are also attractive methods for covalent protein bioconjugation, which has bearings on biocatalysis [104, 107], biochemical synthesis [108–110], therapeutic optimization [111, 112], and vaccine design [113, 114]. For example, enzyme immobilization is an established method for stabilization of proteins that allows recoverability of enzymes in biocatalysis [115–117], and has been demonstrated to improve the efficiency of enzymatic cascades by improving pathway flux [108–110]. Immobilization of such enzymes using ncAA can provide greater control over orientation, which is important for maintaining the activity of many enzymes. Similarly, polymer-protein conjugation is a well-established method for stabilizing therapeutic proteins against thermal or pH stress, proteolytic attack, and improving pharmacokinetic profiles [118–120], but is often accompanied by marked decreases in specific activity associated with imprecise control over location and extent of modification. These conjugates can be enhanced by the greater control and specificity of conjugation afforded by ncAA incorporation and targeting [111, 112, 118]. Finally, virus-like particles (VLPs) have emerged as promising candidates for safe, effective vaccines as well as functionalizable nanoparticles for drug delivery [121, 122]. The surface of these proteinaceous nanoparticles can be “decorated” with a variety of antigens or polymers to improve the generation of adequate immune response to presented antigens or mask immunogenicity of the VLP particle [71, 121]. ncAAs provide bioorthogonal conjugation targets to maintain the integrity of both the VLP and displayed antigens [114, 121].

#### Residue-specific labeling for biotechnology applications

In some cases, residue-specific labeling provides adequate control of conjugation site to maintain sufficient protein activity. For example, Met replacement was used to functionalize a VLP which contained only one Met in each capsid monomer [114]. For cases such as these, in which there are small numbers of accessible residues of a certain type, residue-specific labeling may be sufficient. For proteins in which the N-terminal methionine (fMet) is accessible, a mixture of products may still result due to ncAA incorporation at fMet. Additionally, for applications in which a mixture of conjugation sites in the product is acceptable, residue-specific ncAA incorporation offers a simplistic approach circumventing identification of necessary tRNA synthetases. A disadvantage of this approach, however, is that when multiple instances of a replaced residue are surface-accessible, targeting of the ncAA can still result in a mixture of products modified at different locations and to different extents, similar to that seen with targeting of natural amino acids such as lysine [101]. This limitation is particularly important in development of conjugated proteins for medicinal applications, where consistency in product specifications and performance is key.

#### Site-specific labeling for biotechnology applications

In many applications, including both the study of protein function and the design of enhanced proteins, it is desirable to incorporate the ncAA precisely at a pre-determined site. For example, conjugation site has been shown to have a significant effect on the stability and activity of antibody-drug conjugates [123], polymer-protein conjugates [111, 112, 118], and immobilized proteins [124]. Site-specific ncAA incorporation enables precise control of conjugation site to allow optimization as well as production of homogenous protein conjugates. This homogeneity is especially important for therapeutic applications such as antibody-drug conjugates and polymer-conjugated therapeutics where precise characterization is necessary [70, 111, 112, 123, 125, 126]. Therefore, protein conjugation for biotechnology applications must often be done in a site-specific manner to optimize conjugate homogeneity, activity, and protein stability. For example, using the ncAA acetylphenylalanine (Acf, Fig. 3), polyethylene glycol conjugation (PEGylation) of human growth hormone (hGH) was optimized for conjugation site, enabling mono-PEGylation and development of an active PEG-hGH with increased serum half-life [111]. Notably, Cho and coworkers reported as much as a 3.8-fold increase in the C_max_ of the optimally PEGylated hGH compared to hGH PEGylated at other sites, demonstrating the importance of site optimization and precise conjugation site targeting for pharmacokinetic properties [111].

In biocatalysis and enzyme production, site-specific incorporation of ncAA can be instrumental in preparation of robust, reusable proteins to improve industrial applicability. Deepankumar and coworkers immobilized transaminase to a chitosan substrate site-specifically to produce an immobilized enzyme which facilitated simple purification and maintained a specific activity nearly equal to that of the wild-type enzyme [104]. The enhanced potential for optimization of conjugated enzymes is further demonstrated in a study by Mu and coworkers, in which monoPEGylated derivatives of fibroblast growth factor 21 (FGF21) were prepared via site-specific incorporation of Acf (Fig. 3). This study identified multiple PEGylated derivatives of FGF21, including one where the substituted residue was originally a leucine, which maintained high activity and 15–30-fold increased half-lives [112]. By contrast, another leucine substitution in the same protein resulted in a conjugate which was entirely inactive, highlighting the necessity for site-specific versus residue-specific modifications in maintaining activity of some proteins [112]. These studies emphasize the importance of precise control over conjugation site selection for optimal design and production of biotechnology products such as therapeutic protein conjugates and biocatalysts.

Site-specific ncAA incorporation also allows for close control over the number of sites which are modified by conjugation, which is an important aspect of conjugate optimization. For example, Wilding and coworkers recently demonstrated that dual PEGylation of T4 lysozyme at two site-specifically incorporated Azf (Fig. 3) residues decreased the double Azf-incorporated T4 lysozyme variant’s activity and did not increase its stability, despite increases in the stability and activity corresponding to PEGylation of each site individually [118]. Similarly, close control over conjugation extent for antibody-drug conjugates is necessary to ensure drug homogeneity and enhance therapeutic index [126, 127]. Motivated by this capacity to improve antibody-drug conjugates through close control of drug-antibody ratios (DAR), Zimmerman and coworkers engineered a high fidelity tRNA/aaRS pair to incorporate the highly click-reactive ncAA azido-methyl-phenylalanine (AMF) site-specifically into a Trastuzumab antibody fragment [126]. The researchers demonstrated drug-to-antibody ratios ranging from 1.2 to 1.9 depending on the AMF incorporation site, and potent cytotoxic activity which correlated with the DAR of each variant tested [126]. Recently, Oller-Salvia and coworkers further demonstrated the ability to closely control DAR by using site-specific incorporation of a cyclopropane derivative of lysine to achieve drug-conjugated Trastuzumab with a DAR of > 1.9, indicating high conjugation efficiency of the two ncAA sites within the fragment [127]. Together, these studies illustrate the utility of site-specific ncAA incorporation in biotechnology towards the production of optimized, controlled, and well-characterized conjugates for medicinal and biocatalytic applications.

Given the varied, site-dependent effects of ncAA incorporation and conjugation, a major challenge with ncAA incorporation is understanding and predicting the impact that the mutation will have on the protein. However, recent progress has demonstrated the potential for molecular simulations to inform site selection [118, 124, 128]. For example, simulations unexpectedly predicted a 3% solvent accessible location as being highly stabilizing to the protein if covalently immobilized at this site [128]. Common design heuristics would prevent this site from ever being considered; however, using the ncAA propargyloxyphenylalanine (Pxf, Fig. 3), this site was shown to be better than highly surface accessible sites [124]. Using the same protein, simulation screening was also effective in predicting optimal specific sites for PEGylation, which were different than those predicted for immobilization [118]. The predictions were validated with high correlation using copper-free click-chemistry reactive ncAA Azf (Fig. 3) [118]. Due to these recent successes using molecular simulation, it is anticipated that rapid simulation approaches will increasingly assist in determining the best locations for ncAA incorporation for both the bioconjugation application and for reducing or eliminating structural strain due to the ncAA mutation. As tools for ncAA incorporation continue to increase in efficiency and simplicity and costs continue to decrease, it is anticipated that ncAAs will become not only a research tool for bioconjugation optimization, but also an industrially viable therapeutics and biocatalysts production platform.

**Fig. 7:**
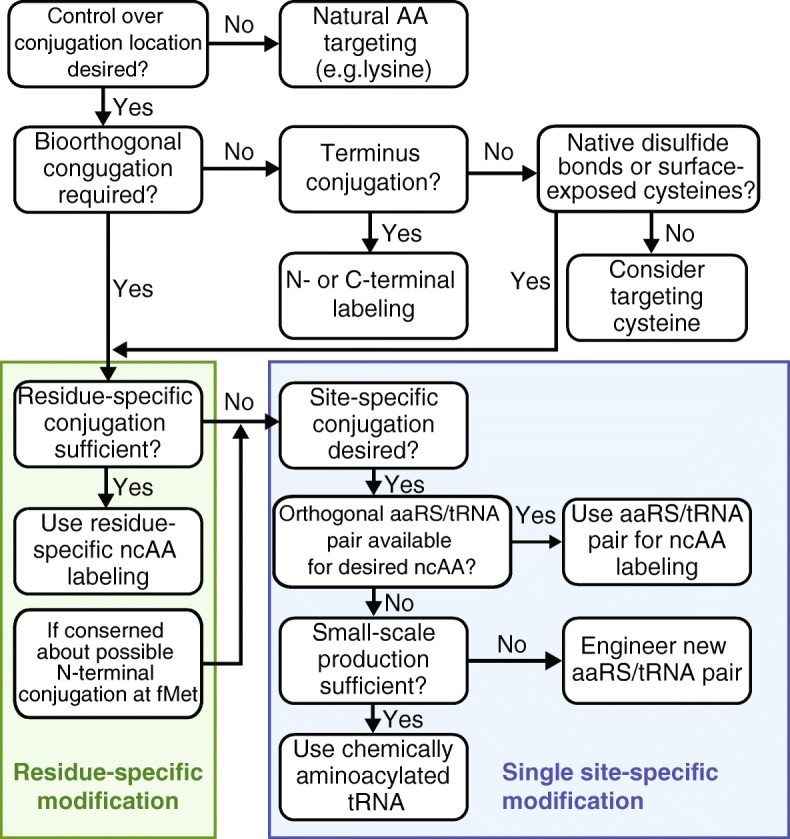
Decision tree for ncAA biotechnology applications. For bioconjugation, it is easiest to target natural amino acids such as lysine, however, this approach provides minimal control over the conjugation site. In addition, the conjugation chemistry is not biorthogonal such that other proteins in the sample will also be conjugated. If biorthogonality is not necessary, the natural N- or C- terminus of the protein can also be targeted. Cysteine can also be targeted, but this can interfere with disulfide bonds if present in the protein. In addition, cysteine conjugation may require some mutagenesis for site-specific conjugation as native surface-exposed cysteines need to be removed and replaced with cysteine at the desired conjugation location. If biorthogonal conjugation is desired and/or greater control over the conjugation site is desired, then first consider residue-specific ncAA incorporation. This has some of the same limitations as targeting natural amino acids as this method replaces a natural amino acid with an analog. However, for proteins with a small number of methionines, this could work well for the desired application. In some studies partial ncAA incorporation at the N-terminus has been observed. If precise predetermined control of the exact locations for conjugation is desired, consider site-specific ncAA incorporation using orthogonal aaRS/tRNA pairs. If aaRS/tRNA have not been engineered to incorporate the desired ncAA for the desired conjugation reaction, chemically aminoacylated tRNA can be used at the small scale. Otherwise, an aaRS/tRNA pair will need to be engineered. Fortunately, a number of aaRS/tRNA pairs have already been engineered for site-specifically incorporating click-chemistry reactive ncAAs

With the increasing variety of approaches to site-specific ncAA incorporation, it is important to identify which approaches are best suited to a given application. Figure 7 provides a decision tree to aid in tool selection based on the needs of a specific application. If bioorthogonal conjugation is not necessary, conjugation at the C-terminus, with cysteine or with other natural amino acids such as lysine, could be considered. However, significant mutagenesis may be necessary to enable site-specific conjugation. In contrast, ncAAs provide bioorthogonal conjugation and facilitate control over the conjugation location with minimal mutagenesis. For proteins in which there are a limited number of surface-accessible instances of a residue such as Met, residue-specific ncAA labeling may be the most effective as it can be done without orthogonal translation machinery. Nevertheless, potential incorporation of a ncAA at fMet must be considered and a site-specific approach should be taken if fMet labeling is a concern. For any site-specific application, orthogonal aaRS/tRNA pairs enable straightforward implementation of nonsense and frameshift suppression, especially for in vivo protein synthesis applications, and are ideal when available. When an aaRS has not been engineered for the desired ncAA, chemically aminoacylated tRNA may be used. However, for large-scale applications, the higher cost of this approach motivates engineering of an orthogonal aaRS/tRNA pair. Finally, as will be discussed in the future directions section, a cell-free protein synthesis approach should be considered in cases where high-throughput evaluation or on-demand production of conjugates is necessary.

## Future directions

To expand the potential of ncAA labeling for research and industrial applications, additional studies are necessary to address key limitations in the efficiency of ncAA incorporation and optimal modification site selection. It is generally recognized that one limitation of residue-specific ncAA labeling is that it commonly requires prior depletion of a natural amino acid to achieve high proteome labeling. This practice can disturb normal biological functions and hence adapting methods that enable high levels of ncAA incorporation in the presence of the canonical amino acid is an important advancement for applications in higher-order organisms [53, 55, 85–87].

Current challenges in obtaining the highest quality proteomic mapping lie in the optimization of the click chemistry reactions and enrichment protocols. Therefore, continued discovery of new click chemistries with faster kinetics and higher specificity will increase the potential for ncAAs in proteomics applications. In addition, development of techniques that allow cell- and tissue-specific labeling in mammalian systems with lower non-specific labeling and background noise will have a significant impact on resolving cellular proteomics maps with high resolution. This, combined with advances in engineering aaRS mutants that enable charging ncAAs at higher rates and promoters that can drive the expression of the mutant synthetase with high cell specificity, will enhance our understanding of the spatial and temporal aspects of proteome dynamics.

A major hurdle for biotechnology applications where stoichiometric labeling is desired is that ncAA incorporation efficiency for site-specific protein modification often varies by the incorporation site. Elucidating factors determining site-dependence will enable the more effective design of ncAA-modified proteins, for example, by targeting bases that flank suppressed codons [129]. Additionally, investigation of mechanisms involved in ribosome stoppage, where polypeptide synthesis stalls or terminates prematurely, may also provide illumination towards efficient modification site selection. Development of novel cell strains lacking factors inhibitory to ncAA incorporation may also improve labeling efficiency. Such strains have already been developed in *E. coli* by knocking out release factor components responsible for competition with nonsense suppression at amber stop codons to reduce premature termination [125, 130, 131]. However, development of such strains for other organisms or ncAA incorporation methods may be challenging as the rarely used amber stop codon required significant mutation before a viable *E. coli* strain was produced [125, 130, 131].

Protein labeling, even site-specifically, can also have a dramatic effect on the properties of the protein in a manner that is highly dependent on modification site/sites. Currently, no complete set of parameters exists to identify sites amenable to labeling based on the primary, secondary, or tertiary structural context [118]. This limitation is compounded by a similar lack of knowledge regarding the effects of locational dependence on ncAA incorporation [118, 129]. In order to capitalize on the benefits of ncAA incorporation for biotechnology applications, tools that enable the rapid identification of sites most amenable to ncAA incorporation and post-translational modification are necessary. Such tools include high-throughput screens for modification site evaluation and development of accurate parameters for ncAA incorporation into coarse-grain molecular models to enable rapid in silico screening of modification sites. Development and refinement of such tools are critical to circumvent costly design/build/test cycles for advanced proteins in fields such as imaging, medicine, and biocatalysis.

Another potential solution to improve ncAA incorporation into particular proteins of interest is in vitro or ‘cell-free’ protein synthesis where some of the factors limiting ncAA incorporation can be overcome. For example, multiple labs have removed the native tRNAs and then added a minimal set of in vitro synthesized tRNAs, essentially emancipating most codons for competition-free ncAA incorporation [63, 132]. Additional advantages that in vitro or ‘cell-free’ protein synthesis provides over in vivo expression include direct access to the reaction environment, eliminating transport limitations of ncAAs across cell membranes and walls and allowing facile supplementation with exogenous components to improve incorporation efficiency [69, 133]. The flexibility of this system allows the incorporation of less-soluble ncAAs with click-compatible side chains, expanding the repertoire for protein labeling [133]. Importantly, cell-free systems can also be lyophilized for on-demand distributed use in an endotoxin-free format for point-of-care medicinal applications or for rapid response to market demands for biochemical products [134, 135].

In conclusion, ncAA labeling is a versatile tool that enables the identification of de novo protein synthesis and proteome dynamics and adds new functionality to proteins of interest. With the continued development of new technologies for ncAA incorporation, it is increasingly difficult to determine the best approach for a given application. To assist in the experimental design of new applications of ncAA labeling, decision tree diagrams are provided for proteomics and biotechnology applications in Figs. 6 and 7, respectively. It is expected that these technologies will continue to expand into other applications areas in proteomics and biotechnology and be used to increase insights into spatiotemporal protein expression patterns, protein structure-function relationships, and to open new avenues into engineering new protein functions.

## Abbreviations

aaRS: aminoacyl tRNA synthetase
Acf: Acetylphenylalanine
Aha: Azidohomoalanine
Anl: Azidonorleucine
Azf: Azidophenylalanine
CuAAC: Copper(I)-catalyzed azide-alkyne cycloaddition
Hag: Homoallylglycine
Hpg: Homopropargylglycine
Met: Methionine
ncAAs: non-canonical amino acids
Pxf: Propargyloxyphenylalanine
SPAAC: Strain promoted azide-alkyne cycloaddition
